# Altered sense of Agency in children with spastic cerebral palsy

**DOI:** 10.1186/1471-2377-11-150

**Published:** 2011-11-30

**Authors:** Anina Ritterband-Rosenbaum, Mark S Christensen, Mette Kliim-Due, Line Z Petersen, Betina Rasmussen, Jens B Nielsen

**Affiliations:** 1 Department of Exercise and Sport Sciences, Panum Institute, University of Copenhagen, Blegdamsvej 3, 2200 Copenhagen, Denmark; 2 Department of Neuroscience and Pharmacology, Panum Institute, University of Copenhagen, Blegdamsvej 3, 2200 Copenhagen, Denmark; 3 Helene Elsass Center, Holmegårdsvej 28, 2920 Charlottenlund, Denmark

## Abstract

**Background:**

Children diagnosed with spastic Cerebral Palsy (CP) often show perceptual and cognitive problems, which may contribute to their functional deficit. Here we investigated if altered ability to determine whether an observed movement is performed by themselves (sense of agency) contributes to the motor deficit in children with CP.

**Methods:**

Three groups; _1) _CP children, _2) _healthy peers, and _3) _healthy adults produced straight drawing movements on a pen-tablet which was not visible for the subjects. The produced movement was presented as a virtual moving object on a computer screen. Subjects had to evaluate after each trial whether the movement of the object on the computer screen was generated by themselves or by a computer program which randomly manipulated the visual feedback by angling the trajectories 0, 5, 10, 15, 20 degrees away from target.

**Results:**

Healthy adults executed the movements in 310 seconds, whereas healthy children and especially CP children were significantly slower (p < 0.002) (on average 456 seconds and 543 seconds respectively). There was also a statistical difference between the healthy and age matched CP children (p = 0.037). When the trajectory of the object generated by the computer corresponded to the subject's own movements all three groups reported that they were responsible for the movement of the object. When the trajectory of the object deviated by more than 10 degrees from target, healthy adults and children more frequently than CP children reported that the computer was responsible for the movement of the object. CP children consequently also attempted to compensate more frequently from the perturbation generated by the computer.

**Conclusions:**

We conclude that CP children have a reduced ability to determine whether movement of a virtual moving object is caused by themselves or an external source. We suggest that this may be related to a poor integration of their intention of movement with visual and proprioceptive information about the performed movement and that altered sense of agency may be an important functional problem in children with CP.

## Background

Although CP is commonly described as a non-progressive disorder of normal sensory-motor development, new research has emphasized that CP also involves alteration of perception and cognitive abilities depending on the site, extent and time during development of the lesion [1,2]. It is conceivable that the lesion may interfere with proper perception and judgment of motor capacity and performance in some children with CP, but this is little investigated. Furthermore, a key element of improvement of motor control and motor capacity is the ability to differentiate self-produced movements from movements originating from the external environment [3-5]. Healthy subjects need only limited attention in order to recognize self-produced movement, whereas subjects with brain lesions, especially within brain regions associated with sensory-motor integrations, are less capable of recognizing the proper agent of a particular movement [3,6,7]. This may have a profound influence on the progress of recovery of function (see Sachs 1984 for a first person description) [8]. It is unknown whether patients diagnosed with CP have a decreased ability to recognize the proper agent of a particular movement (the sense of agency) and to what extent altered sense of agency has an impact on their motor performance. Clinically it would be of great importance to provide thorough information of this aspect in order to address better rehabilitation programs for CP children.

One way of studying the sense of agency is the so-called "Alien hand experiment" [9]. In this experiment subjects were instructed to draw a straight line while observing their hand through an opening in a box. Without their knowledge, a mirror in the box could be manipulated so that they would randomly see an "alien hand" (the experimenter's hand) or their own producing the movement. As long as the subjects viewed movements which were similar to their own intentions, they perceived the movements as being produced by themselves even when they were not the agent of the observed movement [9]. Only when the experimenter's movement deviated significantly from the intentions of the subject (straight line), would the subjects discover that something was wrong. Newer studies have followed the basic principles of the Alien hand-experiment to investigate the neural correlates of agency in patients with brain lesions. In these experiments subjects have been presented with video-images showing either their own hand or the hand of an experimenter performing movements that are either congruent or incongruent with the subjects own movements. It has been found that subjects with lesions of the parietal cortex have a significantly reduced ability to correctly identify whether the observed movements were caused by themselves or not [3,10,11].

The goal of the present study was to investigate whether children with CP may have an altered sense of agency similar to adult patients with parietal lesions and whether this contributes to their motor disabilities. In order to reach this goal we developed a computer-based version of the alien-hand experiment, which permitted evaluation of both the subjective sense of agency and the kinematics of the performed movement. We found that children with CP have a reduced ability to distinguish when an observed virtual object is moved by them or by an external source, and we suggest that this may contribute to their functional disabilities.

## Methods

### Participants

The study was designed as a cross sectional experiment including 24 children (17 boys and 7 girls) diagnosed with spastic hemiplegia CP in the ages of 8-16 years (average age: 11.3 ± 2.0 (SD)). We selected CP children on the basis of medical records. Upper limb function was assessed in the CP children by the Manual Ability Classification System (MACS) [12] which classify the ability of CP children to use their hands separately or together in daily activities. Furthermore, the CP children were also evaluated by the Gross Motor Function Classification System (GMFCS-level) [13] and they completed a Test of Visual Perception Skills (TVPS) consisting of seven different visual perception tests [14]. Overall performance in the tests is given by the sum of each individual test's score scaled according to the normative data for the child's age group and a normalisation score. 17 of the CP children provided medical records with information about their diagnosis. In all children the insult occurred pre-birth. None of the CP children had visual field defect. Table 1 describes the CP children.

**Table 1:** Personal data of the group of CP children

Child	Age (y,m)	Dominant hand (r/l)	Sex	Hemiplegia (Rt/Lt hemiparesis)	Birth weight (g)	Term (w)	GMFCSLevel	MACS	TVPS (sum scaled score)	TVPS (standard score)
CP1	8	r	f	V, Rt	2560	-1	I	1	53	87

CP2	8.11	r	m	V, Lt	1940	-8	I	1	89	113

CP3	8.8	r	f	V	3500	-3	II	1	41	79

CP4	8.9	l	m	V, Rt	3590	0	I	2	59	92

CP5	9.6	r	m	V, Lt	2170	-5	I	2	65	96

CP6	9.4	l	m	V, Rt	3500	0	I	2	57	90

CP7	9.9	l	m	V, Rt	4350	0	I	2	60	93

CP8	10	r	f	V, Lt	4280	+2	I	1	101	122

CP9	10.5	l	m	V, Rt	2805	-5	I	1	102	123

CP10	10.9	l	f	V, Lt	3950	0	I	1	83	109

CP11	10.9	l	m	V, Lt	3800	+2	I	2	67	98

CP12	11.9	l	f	V	3070	0	I	1	53	87

CP13	11	r	f	V, Lt	2800	0	I	1	75	103

CP14	11	r	m	V, Lt	4570	+2	I	1	109	128

CP15	11.2	r	m	V	3600	0	I	1	109	128

CP16	11.6	r	m	V, Lt	4070	0	I	2	91	115

CP17	12.1	r	m	V	1533	-10	I	1	56	90

CP18	12.4	r	m	V	1610	-10	I	1	64	96

CP19	12.7	l	m	V, Rt	4840	0	I	2	50	86

CP20	13.9	r	m	V	1050	-8	I	1	63	95

CP21	13.9	l	f	V, Rt	1542	-8	I	1	70	100

CP22	14.1	r	m	V, Lt	3800	0	I	2	90	114

CP23	14.1	r	m	V, Lt	1648	-9	I	2	47	83

CP24	15.1	r	m	V	3250	0	I	2	33	73

Table 1 describes personal data of the group of CP children included in the study. Personal data (age, dominant hand, sex) is given. Where it was possible, information about their diagnosis, the side of the lesion (Rt:right, Lt:left) and perinatal data (birth weight and prematurity) is listed (negative values indicate weeks of pre-term). Furthermore, the CP children's test scores for GMFCS, MACS and TVPS are indicated.

A control group consisting of 65 healthy children, 37 boys and 28 girls (average age: 11.6 ± 2.2 (SD)) were recruited through contacts with local schools in the area around Copenhagen, Denmark. Furthermore, we incorporated a third control group of 16 healthy adults, 11 males and 6 females (average age: 23.8 ± 3.7 (SD)). The adults were students at the University of Copenhagen, Denmark. Sixty-two of the children and 14 of the adults were right-handed and three children and three adults were left-handed. This was assessed by asking the subjects which hand he/she would use when writing and drawing in accordance to the Edinburgh inventory of handedness assessment [15].

Our pilot studies revealed that children under the age of eight could not fully understand the instructions and they were therefore excluded from the study.

All subjects were given written and verbal introductions regarding the experiment. The participants gave informed consent, and for underage participants, their parents and/or legal guardians provided a written consent form prior to beginning of the data collection. The study was approved by the local ethics committee (protocol number: H-B-2009-17) and the study was conducted in accordance with the declaration of Helsinki guidelines.

### Apparatus and procedure

**Figure 1:**
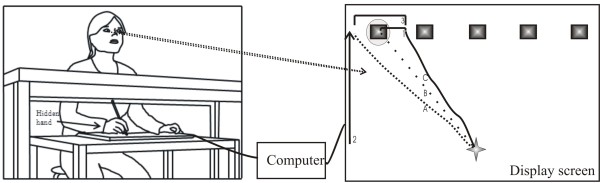
**illustrates the experimental setup**. The display screen shows the 6 objects, which of one is the visual object (in the lower center) and one is the hit target (the object marked with a circle). The pen-tablet and the display screen are connected to a computer, which collects the information about the trajectories of the visual object and the actual produced movement on the tablet. Explanation of the letters and numbers inside the display screen: A: the trajectory of the visual object, B: the direct path of the visual object to the target, C: the trajectory of the actual produced line on the tablet. 1: indicates the hit-distance, 2: illustrates the time it takes to move the visual object to the hit target, 3: specifies the drift of the actual movement (C) on the basis of the trajectory of the visual object (A). There are no lines visible on the display screen during the experiment.

Figure 1 illustrates how the subjects were seated comfortably in a chair 2 meters away from a screen (1.58 m × 1.17 m), which acted as a display screen. Just in front of the subjects a graphic pen-tablet (WACOM, Intuos3, 210 mm × 297 mm) was placed on a table, but underneath a cover in order to prevent subjects from seeing the tablet and avoiding visual input from the arm movement when performing the experiment. The cover did not obstruct the actual movement of the arm/hand as it was elevated to a proper height. All of the subjects used their preferred hand, which they normally use for drawing and writing during the experiment. None of the CP children used their most affected hand, since they would then not have been able to accomplish the task.

Custom-made (programmed in F-sharp) software was used for presentation of the visual object on the computer screen and for data sampling and analysis.

Prior to the experiment, subjects were instructed how to use the stylus on the graphic pen-tablet, and to draw a straight line to the target as quickly and precisely as possible. A few trials were performed preceding the experiment to prevent inappropriate errors in producing the movement, and to make sure the subjects had understood the task. During the experiment they were encouraged to produce the movement as fast and precisely as possible. The reaching movement had to be made in one straight direction at a moderate velocity. In order to move the visual object, the subject had to place the pen on top of the visual object and move the pen towards one of the five targets, which was cued by a coloured circle (cf. Figure 1). If the subjects accidently lifted the pen, the visual object would return to the start position, and the trial would restart. Each trial finished when the visual object reached 180 pixels from the top part of the screen. The targets were cued randomly, and the five targets were changing between 17 different images with a diameter of 200 pixels.

In order to keep the subjects attentive during the test, the subjects were to indicate which of the five targets they were to hit before each trial. During the experiment, the visual object could be moved either by the subject or by the computer program. The manipulations completed by the computer program were given by a straight line with fixed targets of 0, 5, 10, 15 and 20 degrees away from the cued hit-target. A "Free Move" condition was introduced, in which subjects had absolute control of the movement of the visual object. The manipulations of the visual object and the Free Move were randomly introduced with a four time higher rate of Free Move than the other types of movements (5 times of each type of the manipulation and 20 times of Free Move). The purpose of the higher number of Free Move was to allow the same number of free movement as computer-manipulated movements, which deviated from the hit target (corresponding to 5, 10, 15 and 20 degrees). A total number of 45 line-drawings were to be produced by moving the visual object.

The encouragement for the subjects to produce the drawings as quickly as possible was to make all the movements resemble each other in the motor pattern without contaminating the data with hesitant movements, which could arise when subjects experienced incongruence between the actual motor performance and the sensory feedback.

After each trial, subjects had to report whether or not they felt as being responsible for moving the visual object to the target by responding yes or no. They were encouraged to answer as fast as possible thereby not allowing too long time for retrospective consideration of their own motor performance and the visual feedback. All subjects were informed prior to the experiment that they were only responsible for completing the movements in some of the trials.

### Data collection

The data contains the X_pen_, Y_pen _coordinates for each individual movement produced by the pen on the tablet and the X_screen_, Y_screen _coordinates of the visual object on the screen. Both data coordinates were sampled at 60 Hz. Each complete set of coordinates X,Y_pen _and X,Y_screen _corresponds to values illustrated in Figure 1.

All the data containing distance measurements are normalized to the size of the pen-tablet.

**1**) Hit-distance: corresponds to the distance between the hit-target and the end position of the X_pen_, Y_pen _coordinates (mm) (1).

**2**) Time: indicates the time to complete the movement (mm) (2).

**3**) Curvature (*C*); the curvature is given by:

$$c=\frac{x^{\prime }y^{\prime \prime }-y^{\prime }x^{\prime \prime }}{{\left({x{}^{\prime }}^{2}+{y{}^{\prime }}^{2}\right)}^{3∕2}}$$

We used the absolute summed values given by the *C *for each data point (mm^-1^) (3).

**4**) Drift: this is the Eucledian distance between the X_pen_, Y_pen _and the X_screen_, Y_screen_.

$$drift=\sqrt{{\left(x_{pen}-x_{screen}\right)}^{2}+{\left(y_{pen}-y_{screen}\right)}^{2}}$$

This result represents how much the subjects tried to compensate the deviation of the moving object during the course of the trajectory to the target (mm) (4).

**5**) Subjective assessment of reporting "Yes" or "No" of being responsible for the movement.

Only information of line drawings produced in completed movements were stored for later analysis, thereby excluding data information regarding unfinished lines due to lifting of the pen or drawing out of area on the pen-tablet.

### Data analysis

All of the data are presented in population_mean _values. All data analysis was organised off-line after the experimental session. We excluded all data below 20% and above 70% of the display screen to avoid data contamination which could appear when the subject were placing the pen on the table to "pick up" the visual object and when they finished the lines. Furthermore, we excluded trials where the curvature exceeded three standard deviations compared to the average of the line produced by the subject within the specific manipulations of the visual object's trajectory or if the X_pen _Y_pen _coordinates for individual trials were insufficient. This corresponded to a total of 4.3%, 4.7% and 7.9% of trials from the group of adults, the healthy children and the CP children, respectively.

### Statistics

Statistical analyses were performed using SigmaPlot software (version 11). The population mean, standard deviation and the Standard Error of the Mean (SEM) of observation within each population group were calculated for each individual motor parameter (hit distance, time, curvature and drift) for the number of observation as well as the subjective assessment score. We used 2-way-ANOVA repeated measures for the factors; Movement manipulation; 6 (free move, 0, 5, 10, 15, 20 degrees) × Groups; 3 (healthy children, CP children, healthy adults). In case of interactions between the factors we proceeded with a post hoc analysis for multiple comparisons using Holm Sidak correction. The statistical differences are therefore reported as the results from the post hoc tests.

For comparison between the subjective assessments we used χ^2 ^for each individual movement type.

The level of significance was set to p < 0.05.

The graphs are displayed as averages across groups with the SEM.

## Results

### Movement kinematics

**Figure 2:**
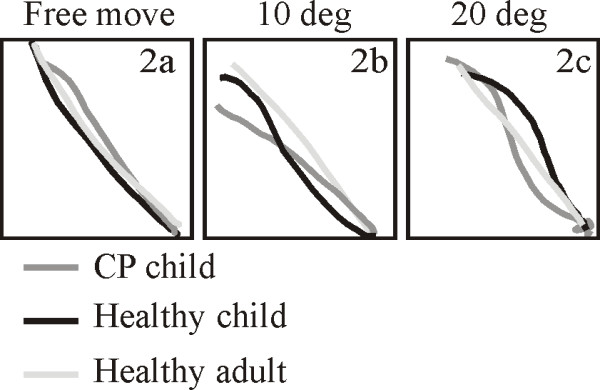
**Single trial comparisons between the subject groups of the actual produced movement**. The figure represent examples of individual drawings of the produced movement when the visual object is; free move, 10 and 20 deg manipulations when cued to the target in the upper left corner. The single trials within the CP group, is taken from an 8 and 9 year old CP child. The single trial examples of movements for the group of healthy children, is given by an 8 and a 12 year old healthy child.

All children and adults were able to perform the requested movements, but with a clear difference in the performance level among subjects as seen when comparing lines drawn by an 8 or 9 years old CP child, 8 and 12 years old healthy child and healthy adults (cf. Figure 2a, b, c). Although all three subject-groups hit the target quite well during the self-generated movement and at all the movement manipulations, the lines drawn by the adults and healthy children were less curved than compared to the children with CP (F[2,498] = 8.708, p < 0.01). It is also seen that especially the adults reacted more promptly when the object deviated from the straight line to the target in the trial with 20 degree deviation than the children with CP.

**Figure 3:**
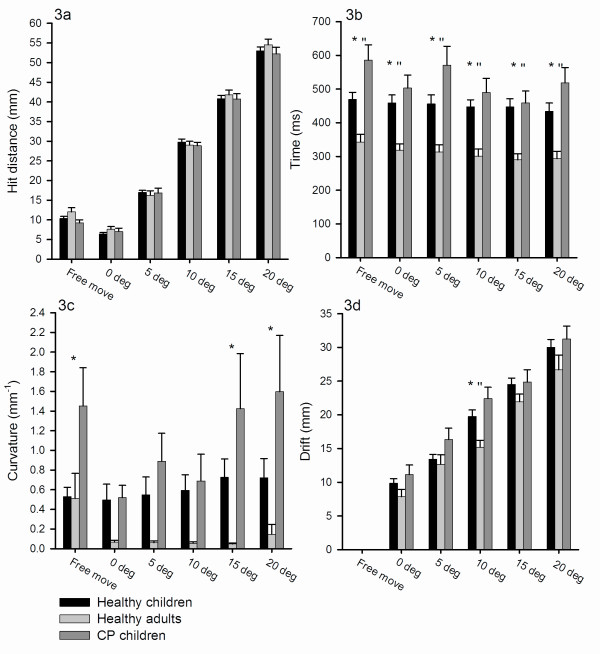
**Motor performance comparison for the three experimental groups**. Figure 3a displays the average distance (mm) for the hit-distance for the three groups in the different categories of movement manipulations. There is no statistical difference between the three groups (p = 0.389). 3b depicts the averaged time (ms) it takes for the three groups to complete the different movement manipulations. The * represents a significant (p < 0.03) difference between the CP children and one or both of the healthy groups and the "represents the significant difference between the healthy children and the healthy adults. 3c illustrates the curvature (mm**^-1^**) for the three groups during the different movement manipulations. The * and "represent significant difference between the CP children and the two healthy groups and the healthy children and the healthy adults (p < 0.003). The greater the curvature is, the more the deviations from a straight line occur for the visual moving object to reach the target. 3d shows the average drift (mm) for the three groups during the individual movement manipulations. The drift is a measure of how much the subjects are deviating or compensating for the computer manipulation of the visual objects in the reverse direction. As there is no computer manipulation at Free Move, the Drift is zero. The greater the score, the more the subjects have tried to compensate for the deviation of the visual object. The * represents a significant difference between the CP children and the both of the healthy groups, and the "indicates a significant difference the healthy adults and the healthy children (p < 0.05).

The findings illustrated in Figure 2 for single subject's line drawings were confirmed when pooling data from all subjects in the three groups. Figure 3a shows that the hit distance (the absolute distance between the final position of the pen to the center of the target) did not vary much between the groups for the 5 different computer-generated manipulations of the visual object and the self-generated movement The interaction between groups and movement type provides no statistical difference (F[10,498] = 0.579, p = 0.832). Unsurprisingly, the distance between the pen and the target increased with the introduced deviation of the line. All groups thus corrected the observed deviation of the object to the same extent when measured as the final position of the pen.

Although there were thus no differences in the hit distance, clear differences in the other measures characterizing the trajectories of the movement before reaching the final position between the populations were found. Most importantly, children with CP were slower in performing the movements than the other two subject groups as seen from Figure 3b. There were no significant differences in the movement time whether the subjects were responsible for the movement of the visual object or whether the computer introduced any of the deviations and data from all movements were therefore pooled for the three groups. The analysis showed that children with CP used significantly longer time than the adult subjects (543 ms as compared to 310 ms; (p < 0.001). They also used statistically longer time than the healthy children (456 ms as compared to 543 ms) (p < 0.04). There was also found a statistically significant difference in the movement time for healthy children and healthy adults (p = 0.05) (F[2,498] = 9.480, p < 0.001).

There was a tendency for the curvature of the produced line (corresponding to line a, see Figure 1) to be larger when the computer introduced larger deviations of the visual object for the CP group, but this did not reach statistical significance for any of the groups (Figure 3c). When pooling data from all movements the post hoc comparisons tests showed that the curvature was found to be significantly longer for the CP children as compared to the healthy children (1.26 mm^-1 ^as compared to 0.62 mm^-1^; p < 0.003) and healthy adults (0.15 mm^-1^; p < 0.001). There was no significant difference in the curvature between the healthy children and healthy adults.

The drift of the produced line (corresponding to the subjects' immediate response to the deviation of the observed object, (see Figure 1, number 3) increased significantly with the size of the deviation of the observed object for all three groups (Figure 3d). Post hoc comparison of interaction between the groups and the movement types showed that the drift in general was larger for children with CP than for the other two groups, but this only reached statistical significance at 10 degrees (p < 0.001). A statistically significant difference between healthy children and adults was also observed for 10 degree manipulation (p = 0.013) (F=[2,498] = 3.431, p < 0.036).

### Subjective report of agency

**Figure 4:**
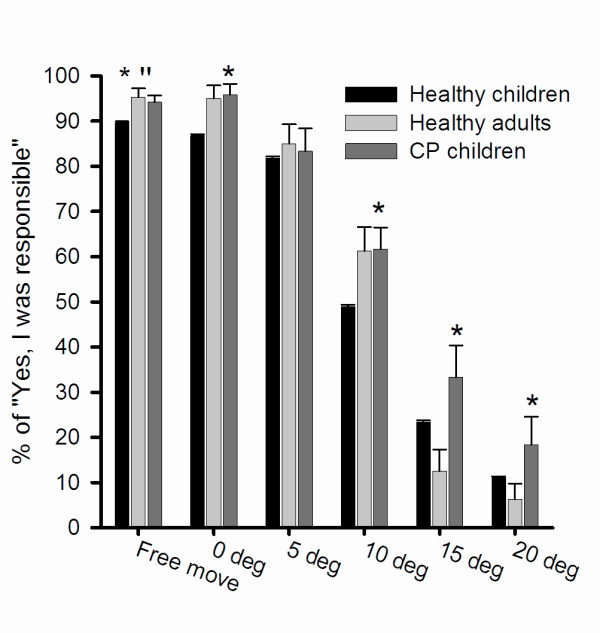
**Subjective assessment of agency for the observed movement**. The graph displays the percentage of the groups reporting "Yes" according to what the subjects estimated were their responsibility for moving the visual object in the individual movement manipulations. The * indicates the significant difference between the CP children and either one or both of the healthy groups (p < 0.05) and the '' indicates as significant difference between the two healthy groups (p = 0.05).

All subjects significantly more frequently reported that they were not responsible for the observed movement of the object, the larger the deviation of the observed object (Figure 4; p < 0.05). The CP children reported significantly more often that they felt as being responsible for the movement of the visual object compared to one or both of the two healthy groups (p < 0.05) except for the 5 degrees movement manipulation. There was no difference between the healthy adults and healthy children in the reporting-rate expect for the "Free move" movement (0 < 0.05).

### Correlations

For free movements, the closer the end point of the pen was to the selected target, the more often the subject reported that they themselves were responsible for the movement of the object (p < 0.001). We did not find any correlation between age and any of the kinematic measures nor between the age and the subjective report of agency among the healthy or CP children (Spearman ranked order correlation: p > 0.05).

**Figure 5:**
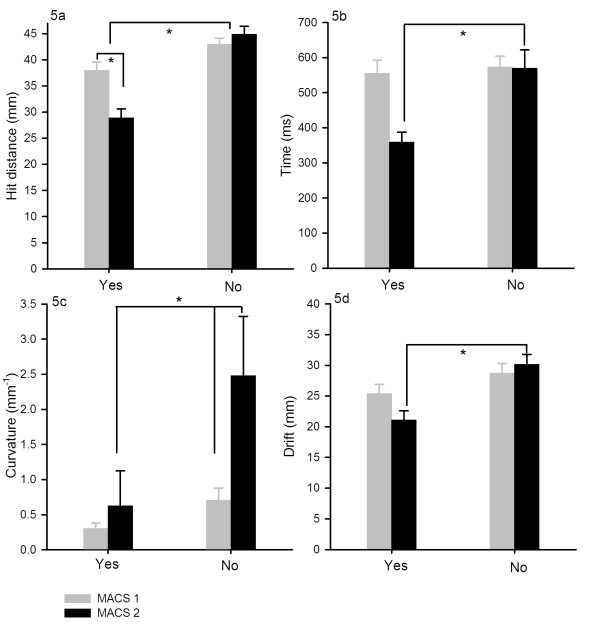
**Comparison of motor performance parameters in relation to the CP children's MACS score**. Figure 5a, b, c and d depicts the responses for the pooled data from the computer manipulations 10, 15 and 20 degrees divided into the MACS score groups for the CP children. Only the functional measurements where we found a significant difference between the CP children and the healthy groups are displayed. Significant differences between the MACS score and the motor performance parameters (time, curvature and drift) was observed for their responses of subjective reporting of being responsible for the visual object or not (p < 0.033). For time and drift measurements, there is a significant difference between the two MACS groups when reporting either Yes or No, whereas there is a significant difference between the reporting rate depending on the amount of curvature for both MACS score groups.

For 10, 15 and 20 degrees deviations a significant difference between the MACS score and the motor performance parameters (hit distance, time, curvature and drift) for the CP children was observed (p < 0.039). Figure 5 illustrates that CP children with high MACS scores were significantly worse than CP children with lower MACS scores in determining whether they or the computer was responsible for the observed movement.

**Figure 6:**
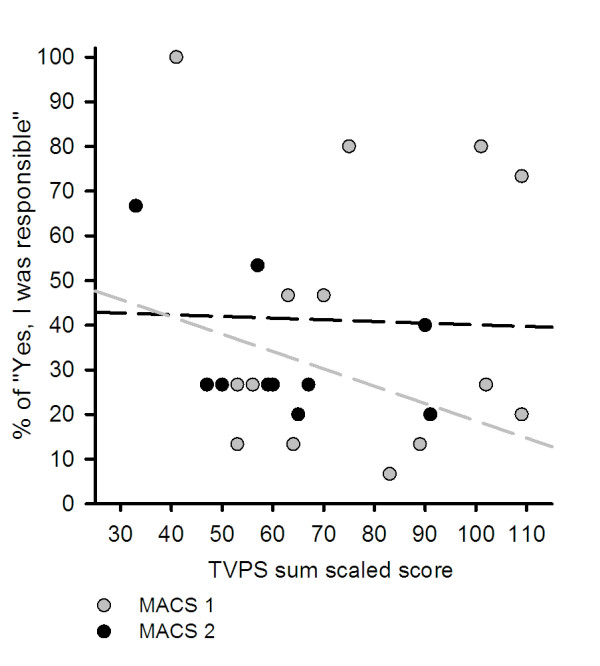
**Correlation between the TVSP scores and the reporting of being responsible for the movements**. Figure 6 illustrates the correlation between the TVPS scores (x-axis) of the CP children and pooled data of 10, 15 and 20 degrees computer manipulations of the percentage reporting of "Yes" for being responsible for the movement. The graph compares the CP children divided into the MACS score groups. r**^2 ^**scores for MACS 1 and MACS 2 are 0.2 and 0.0007 respectively.

We found no significant correlation between the CP children's visual perception scores and their ability to report whether they were responsible for moving the visual object on the display screen or not for the pooled 10, 15 and 20 degrees deviations. The correlation coefficient for the relation between the TVPS score and the % of "Yes" reports was 0.05 for the population of CP children. Splitting the data up in children with MACS score 1 and 2 gave correlation coefficients of 0.02 and 0.0007 respectively (Figure 6).

## Discussion

The present study has shown that CP children falsely attributed computer manipulated movements of an object as being caused by themselves more often than healthy children or adults did. As a consequence, CP children also more often attempted to counteract the movement of the visual object causing a larger discrepancy between their own movement and the computer generated movement of the visual object. We have also shown that CP children perform the movement with a slower time to complete the movement and a longer trajectory given by a greater curvature of the actual produced movement compared to the healthy groups. Finally, there was a relation between the subjective assessment and the motor disability of the CP children. We suggest that this may be related to a poor integration of their intention of movement with visual and proprioceptive information about the performed movement and that altered sense of agency may be an important functional problem in children with CP.

### Is the method for testing agency valid?

The basic idea of the study was to develop a computer-model for evaluation of the sense of agency in children with CP. The paradigm that we have used was inspired by the work published by Nielsen [9]. In his experiment, subjects were asked to draw a straight line on a piece of paper without knowing that their visual information about the movement of the hand was sometimes manipulated to show the movement of the experimenters hand instead. The experimenter's movement could be presented either congruently or incongruently with the subjects own movement. Often subjects, believing that they were the agent, would attempt to correct the movement when the (seen) movement by the experimenter deviated from the (unseen) movement of the subject. Only after several trials would most subjects discover that they had been tricked.

Other researchers have also adapted this paradigm in order to investigate the sense of agency in patients with posterior parietal cortex (PPC) lesions, schizophrenia and other psychological diseases [3,4,10,16]. In their experiments subjects were shown a video of their own moving hand. But unknown to them, the video image was sometimes exchanged to that of the experimenter's hand performing movements congruent or incongruent with the subjects' own movement. Both subjects with PPC lesions, schizophrenia and other types of psychological disorders were found to have an impaired ability to identify when the observed movements were caused by themselves or by the experimenter [3,4,6,16-18].

One problem with this approach is that it is difficult to quantify how large a deviation between the movement performed by the subject and the movement performed by the experimenter is necessary in order for the individual subject to lose the sense of agency. Another problem is that it is necessary to terminate the experiment when the subjects discover that he has been tricked. This may happen at different times and be caused by various factors in different subjects.

A major aim with the present method was therefore to develop a more objective and quantifiable measure of the sense of agency. We consequently chose to make a computer-based paradigm in which deviations between the observed movement and the subjects' own movement could be systematically varied thereby allowing estimation of the angle of deviation at which the sense of agency was lost. Therefore, subjects in our study were informed from the beginning that the trajectory of the visual object was sometimes caused by them and sometimes by the computer. This also allowed a quantification of the number of trials in which sensation of agency was experienced at the different deviations. Because of this, the estimation of sense of agency was based on continuous introspection of the subjects during the trial rather than their spontaneous reaction to the trajectory of the observed object. They may therefore have used different conscious strategies in the evaluation. The fact that they were allowed some seconds to respond after each trial may have contributed to this. However there was a good correspondence between the subjective report and their spontaneous reaction during the experiment to the sudden deviation of the visual object imposed by the computer, indicating that the subjects reliably reported their experienced sensation of agency. Their decision on the sense of agency may have been influenced by previous choices and experience with the behaviour of the visual object in relation to their own intentions. However, the influence of this was minimized by a short training session prior to the actual experiment. There was in addition no difference in the number of Yes and No responses when comparing the initial and last parts of the experiment.

### What does the method tell us about agency?

Healthy adults and healthy children correctly reported that they were responsible for the movement of the visual object during free movement-trials (> 90%) and they also correctly indicated that they were not responsible for the movement when the visual object deviated by 15 and 20 degrees from the target (< 25%). Despite of the uncertainty due to the knowledge that the computer might in some cases be responsible, subjects in general thus accurately attributed the observed movement as being caused by themselves or by the computer. This is in line with several recent studies, which have concluded that as long as the result of a particular movement corresponded with the intention to perform that movement, the subjects feel as being responsible for the movement [9,19,20]. The sense of agency in all the groups is likely caused by the match between the intention of movement and the outcome of the movement given by the sensory feedback, namely the proprioception and the vision. Subjects also attributed the observed movement to themselves when it deviated by up to 10 degrees from the target. Small distortions of the visual object have been shown to be unconsciously automatically corrected during the reaching movement [21]. This may explain why subjects did not notice that the computer was responsible for the movement in the trials with 0 and 5 degrees manipulations, although, Jakobson and Goodale (1989) argued that distortions have to be less than 3 degrees for unconscious adaptation of the motor output. However, factors such as target and object size may play an important role in the amount of distortions required to influence conscious perception of the deviations.

When the visual object deviated by 10 degrees from the target, subjects were uncertain and (incorrectly) attributed about 50-60% of the movements to themselves. Evidently, a 10 degrees deviation is sufficient for the subjects to notice a discrepancy between their own intention and the observed movement, but insufficient to clearly attribute the movement to the computer. This appeared to be the case for all subjects regardless of their age. We thus found no age-related difference in the sense of agency for the tested age groups. This is consistent with previous findings which have suggested that the sense of agency is established prior to the age of eight years [22,23].

The CP children differed from healthy children and healthy adults by wrongly attributing a higher percentage of trials to themselves rather than to the computer. In line with this, children with CP also attempted to compensate the movement of the visual object more than any of the other subjects (*cf*. Figure 4). Evidently, children with CP have difficulty determining whether a visual representation of a movement corresponds to their own intention of movement and their actually performed movement. CP has been shown in recent years to involve, in addition to the well documented motor symptoms which characterize the disorder, disturbances of sensation, perception and cognition, all of which are important factors for the establishment of sense of agency [24]. Furthermore, the disorder is often assumed to cause a lower self-concept of own physical performance compared to healthy peers, although there is no general consensus in the literature [25,26]. Research related to self-perception within children has provided strong evidence that a certain level of cognitive abilities are required to determine accuracy of self-perception and self-awareness [27]. The inability of the CP children to correctly determine that the computer was responsible for the movement at 10, 15 and 20 degrees deviations may therefore be explained both by perceptual and cognitive and motor problems [9,16,19,28].

We do not have detailed information about the perceptual cognitive and motor abilities of the CP children. The TVPS score varied considerably between the CP children suggesting impaired visual processing in several of the children. However, no correlation between the TVPS score, their sense of agency or their performance of the task was observed suggesting that impaired visual processing was not responsible for the observed problems with their sense of agency and/or performance of the drawing task. All of the children were classified in group I or II of the GMFCS for CP and were relatively well functioning and homogenous. However, children in MACS group 2 performed worse than children in MACS group 1 in determining whether the computer was responsible for the movement. There was thus a good correspondence between the MACS score and their performance during the task. Children in MACS group 2 also showed a significant difference in the subjective assessment of the movement. This suggests that reduced sense of agency may contribute to the motor disability in CP children. The CP children used almost twice as much time to complete the movement than the healthy adults and healthy children. Instead of making a straight line to the target they often made several deviations and online adjustments so that the trajectory of the produced line was significantly longer than in the two healthy groups. Part of this may be directly related to their impaired sense of agency since they were also observed to be more often fooled by the computer generated movement than the other two groups. As a consequence, they more often attempted to compensate the observed movement of the visual object by moving their hand in the opposite direction to the computer thereby causing a longer trajectory and longer movement time. However, it is also likely that the sensory motor impairment of the CP children was the primary reason for their longer movement time and trajectory and that this may as a secondary consequence have influenced their sense of agency. Children with CP are likely to often experience that their actual perceived movements do not fully correspond to their intended movement due to both motor and sensory impairments. As a consequence they may misjudge their own physical abilities. This is in line with studies in healthy and CP children aged 6 to 13 years, which have shown that the lower the self-perception the lower the score of self-reported performance [27,29].

Although we cannot distinguish different movement strategies based on the available data, the fast straight movements performed by the healthy adults and to some extend also by the healthy children suggests that they utilized well-learned feed-forward motor programs, whereas the slow unsteady movements performed by the CP children suggests that they were forced to use a feedback strategy in which the movement had to be corrected repeatedly based on the available visual feedback [30-32]. This likely reflects the sensory motor impairment of the CP children and their inherent insecurity in performing skilled movements. We suggest that reasons for the slower time to complete the movement might be related to the requirement to reach the correct target (the CP children were indeed as good as the other two groups in hitting the right target). Fitt's Law predicts the time required to rapidly and accurately move to a target area: the more time spent on completing a movement, the more precise the result will be [33].

### What brain areas are involved in agency perception?

Patient studies and brain imaging studies suggest that it might not be one region only which is activated when experiencing the sense of agency or lack of agency. Rather it seems as if the networks connecting frontal motor areas (including preSMA) with parietal cortex, (especially inferior parietal cortex) [3,5,34,35] have an important role in the establishment of the sense of agency. A study by Fink et al. (1999) investigated in healthy subjects the neural correlates when experiencing incongruence between sensorimotor states, as the visual feedback was distorted during a movement. They concluded that ventral right lateral prefrontal region was primarily activated by discrepancies between signals from the sensory systems (vision and proprioception). The study by Leube et al (2003) and Nahab et al. (2010), who also used a delayed video images experimental paradigm, supports the idea of an activation of a broad neural network when subjects detect mismatch between the intentional, sensory and motor feedback integration. Unfortunately, we do not have images of the CP children's brain lesions to fully describe the place of their lesions and therefore we cannot determine to what extend their lesion involves these networks. However, this seems likely since several studies have associated loss of white matter in the parietal areas of the brain in children with CP with lower cognitive abilities, especially visuo-perceptive impairments [2]. We did not find a statistically significant correlation between agency perception and the CP children's TVPS scores, suggesting that a general deficit in visual perception as such is unlikely to be the course of their reduced ability to detect whether the movement was caused by themselves or the computer. Children with lesions primarily in the left or right hemisphere as judged from their clinical symptoms were almost equally represented in the dataset (table 1). Children with right hemisphere lesion did appear to have more difficulty in determining whether they or the computer were responsible for the movement, but the small size of the dataset did not permit a statistical comparison. Sirigu et al. (1999) also associated right parietal lesion to reduced agency perception [3].

It also seems likely that some of the CP children in the present study may have had lesions to various extend involving the premotor cortex and associated networks. Future studies combing behavioural assessment and neuroimaging is necessary in order to clarify which networks are involved in the altered sense of agency that we have reported here in children with CP. Regardless of this, our data indicate that the sense of agency maybe an important factor to take into account when evaluating perception and motor deficits in children with CP.

## Conclusion

We conclude that children diagnosed with CP have a reduced ability to determine whether movement of an object is caused by themselves or an external source, thereby altering their perception of agency. We suggest that this may be related to a poor integration of their intention of movement with visual and proprioceptive information about the performed movement, and that altered sense of agency may be an important functional problem in children with CP.

## Competing interests

The authors declare that they have no competing interests.

## Authors' contributions

ARR, MSC and JBN participated in the discussion and design of the experiment. ARR carried out all the experimental work. Data analysis was done by ARR, MSC and JBN. ARR and JBN wrote the manuscript. MKD, LZP and BR provided baseline data of the CP children included in the study. All authors read and approved the final manuscript.

## Pre-publication history

The pre-publication history for this paper can be accessed here:

http://www.biomedcentral.com/1471-2377/11/150/prepub
